# Research on the Prediction Method of Braking Rotation Angle for Remote-Controlled Excavator

**DOI:** 10.3390/s23156780

**Published:** 2023-07-28

**Authors:** Yutong Lin, Jingqi Xiong, Wenlian Zhu, Rui Sun

**Affiliations:** Mechanical and Electrical Engineering, University of Electronical Science and Technology, Chengdu 611731, China; yutonglin@std.uestc.edu.cn (Y.L.); jqxiong@uestc.edu.cn (J.X.); 18982787109@163.com (W.Z.)

**Keywords:** remote-controlled excavator, the center of gravity position, rotational inertia, braking torque, braking rotation angle

## Abstract

To calculate, analyze, and predict the rotation angle during the deceleration and braking process of large remote-controlled excavators, this article established a spatial coordinate system based on a simplified model of a hydraulic excavator’s upper structure. Using the D-H parameter method, a mathematical model of the working device’s center of gravity and its rotational inertia was established. Based on the characteristics of the excavator’s hydraulic system and the relationship between brake torque variations, a prediction model was developed to forecast the stopping position (brake rotary angle) of the excavator’s bucket after braking. Subsequently, the predicted results were validated using simulation and compared with existing experimental data to assess the accuracy of the model. The findings demonstrate that the predictive model exhibited high precision with minimal error. The utilization of this model enabled effective forecasting of the excavator’s braking position changes, providing a theoretical foundation for the intelligent remote control of excavators.

## 1. Introduction

Excavators, as one of the most prominent and widely used engineering machinery, play a crucial role in energy exploration, infrastructure construction, and emergency relief [1]. They have also gradually become indispensable equipment in fields such as transportation, energy, and national defense. However, with the increasingly complex working environment and specific working conditions, modern excavators have put forward stricter requirements in terms of personnel safety, construction accuracy, and work efficiency so that traditional excavators can no longer meet the development needs of future society. Therefore, research on intelligent technology [2], energy efficiency improvement technology [3,4], comfort and safety technology [5,6,7], and environmental protection technology for excavators is of great practical significance. Among them, the development of intelligent technology for excavators is of paramount importance, mainly including autonomous control [8,9], automatic navigation [10,11], and automatic scheduling of excavators. For example, through machine learning and other technologies, excavators can recognize and adapt to complex terrain and obstacles [12,13,14], achieving more efficient operations.

Under normal circumstances, excavators work in harsh environments and even in dangerous places [15]. Operators face the problems of high labor intensity and high safety risks [16]. Remote-controlled and unmanned operation modes have become feasible solutions to these problems and have become the main direction of intelligent excavator development [17]. Among them, the remote-controlled operation mode has been widely used [18]. For example, China Railway Engineering Group used remote-controlled excavators to improve construction quality and efficiency in railway tunnel projects, while the ECR235CL excavator of Swedish company Volvo can be equipped with a remote-controlled system to allow operators to work in dangerous locations. In this mode of operation, the driver needs to operate according to the parameters, such as video and image, that reflect the real-time working conditions and the environmental status returned by the site. However, due to factors such as signal transmission methods, transmission distances, and network bandwidth, the required signals may be delayed [19]. If satellite networks or mobile networks are used for transmission, signal delays may be between 100 and 300 ms [20], while when using dedicated radio communication systems or LANs, this delay can be reduced to 10~50 ms [21]. It should be noted that even in the case of short signal delays, due to the limitations of human physiological conditions, operators still need a certain amount of time to react after receiving the signal, which is mainly manifested as inaccurate positioning or delayed actions [22].

To address the above issues, one approach is to use technological means such as 5G communication to reduce transmission delay [21], while the other approach is to predict relevant operational information under the current signal delay and provide operational assistance to the remote operator [23]. As the hydraulic excavators’ working (rotation) process is not a fixed procedure, it is not possible to use a consistent empirical value to deal with real-time updates of the operational environment and task status. Instead, a relatively general set of variable rules needs to be determined to analyze and calculate relevant parameters. This article took medium-sized hydraulic excavators as the research object and focused on the prediction problem of relevant operational information under the current signal delay, specifically the prediction of the bucket stop position [24] (i.e., the braking rotation angle of the excavator) after the entire vehicle brakes and rotates in the remote-controlled operation mode. By simplifying, decomposing, fitting, and restoring the excavator’s upper structure, a corresponding spatial coordinate system was established to determine the initial center of gravity position of each component of the excavator in the static state. The starting state was then iteratively analyzed and calculated to determine the dynamic changes of the center of gravity position and rotational inertia of the excavator’s upper structure during the relative rotation of the working device and the rotary platform around the swing axis. The braking rotation angle of the vehicle itself was predicted in combination with the torque changes of the engine and the reducer during the rotation process, providing a theoretical basis for the positioning of the bucket for the remote operators and exploring a new method for analyzing the dynamic operation of construction machinery. It also provides a new direction for the intelligent development of construction machinery.

**Figure 1:**
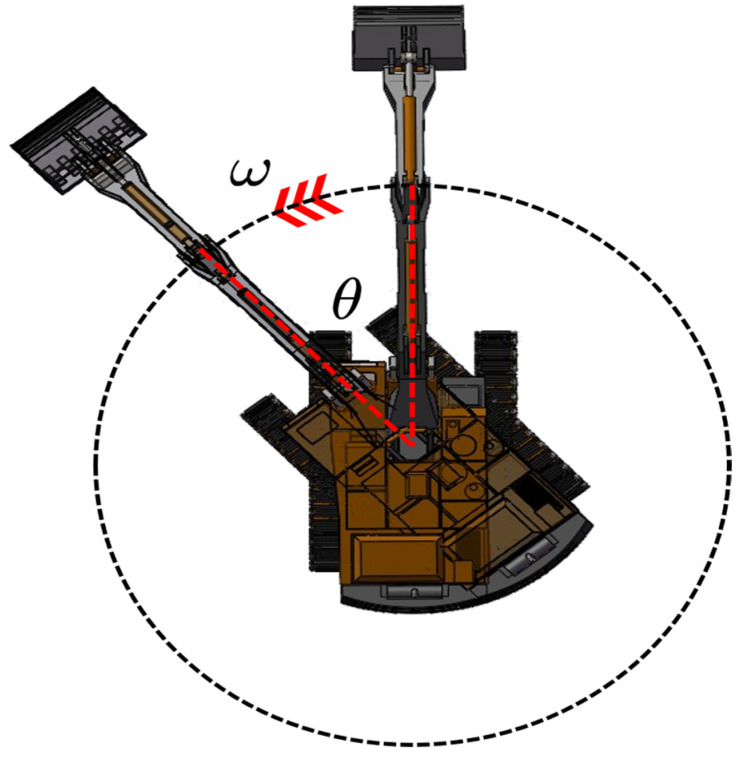
Excavator braking rotation angle.

Figure 1 shows a schematic diagram of the excavator’s braking rotation angle, where ω represents the angular velocity of the vehicle during the rotation process, and θ represents the braking rotation angle, which is the angle between the current position and the predicted stopping position.

## 2. Materials and Methods

### 2.1. Decomposition and Simplification of Excavator Modules

To analyze the dynamic changes in the center of gravity and rotational inertia of the hydraulic excavator during the rotation process, the first step was to determine the center of gravity coordinates of each component involved in the rotation. The commonly used method is to install corresponding sensors on the specific excavator, and then use related algorithms and software for measurement and reading. However, the noise, temperature, and humidity on the construction site can greatly affect the accuracy of the sensors. Therefore, this paper attempted to use a method based on the spatial positions of each component to determine their center of gravity coordinates. The key was to determine a parameter set that can characterize the size of each component of the excavator, to effectively screen and express the data points of the 3D structure. Based on this, mathematical methods can be used to reconstruct its 3D structure and obtain the initial center of gravity position.

#### 2.1.1. Decomposition Module Hinge Point Coordinate Collection

**Figure 2:**
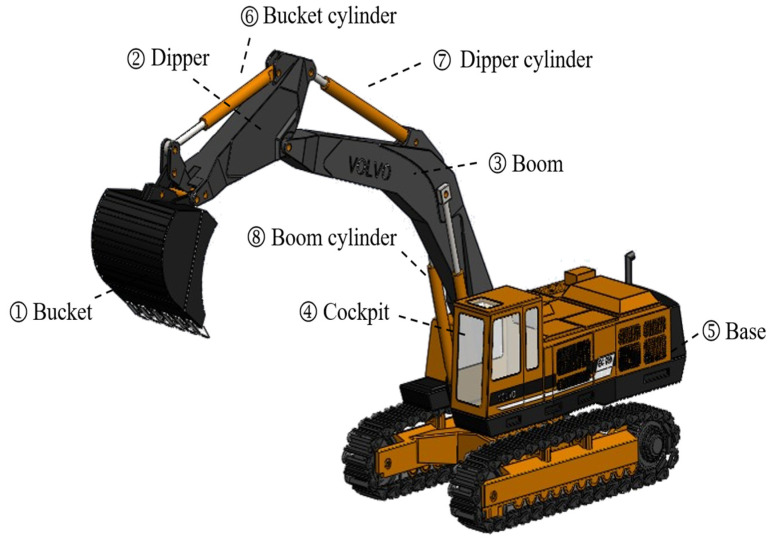
Volvo EC650 excavator Solidworks 3D model.

The Volvo company’s EC650 medium-sized hydraulic excavator was selected as the research object, and the components involved in the rotary motion were identified, including the bucket, dipper, boom, cockpit, base, bucket cylinder, dipper cylinder, and boom cylinder, as shown in Figure 2. The maximum lifting state of the excavator (where each working device reaches the highest and farthest position) was chosen as the initial state.

The three-dimensional structure of the model shown in Figure 1 was converted into effective data points. Firstly, the excavator’s upper structure was disassembled, and each module was a homogeneous component made of steel material. Secondly, we defined feature points as points that can accurately or approximately form the external contour of the model by connecting them one by one. The edge points of the model can be selected as the initial feature points, if it is a plane, they can be directly connected, and if it is a curved surface, the corresponding curvature needs to be determined. We collected the initial feature points of the disassembled modules and organized their spatial coordinates, as shown in Table 1.

The center of gravity coordinates of a three-dimensional model can be directly read using Solidworks. However, the calculation accuracy of the algorithm used by the software was affected by the modeling method of the model. When the geometric shape, mass distribution, or material density of the model changes, manual adjustments and corrections must be made. Obviously, different brands and models of excavators correspond to different models. In practical use, only the center of gravity coordinates of simple objects are calculated through software, and other methods are needed to assist in calculating the center of gravity coordinates of complex and asymmetric objects. In order to calculate the initial center of gravity of the excavator, interpolation based on the initial feature points can be used, that is to predict the values of unknown data points using known data points, thereby expanding the number of effective data points to ensure the accuracy of the calculation result. In theory, the more initial points collected, the better the corresponding interpolation effect. Considering that most of the components in the excavator structure can be represented by straight lines to describe their surfaces or contours, linear interpolation can also achieve good results for data conversion.

#### 2.1.2. Simplification of the Upper Structure and Composite Position Model of Oil Cylinder

**Figure 3:**
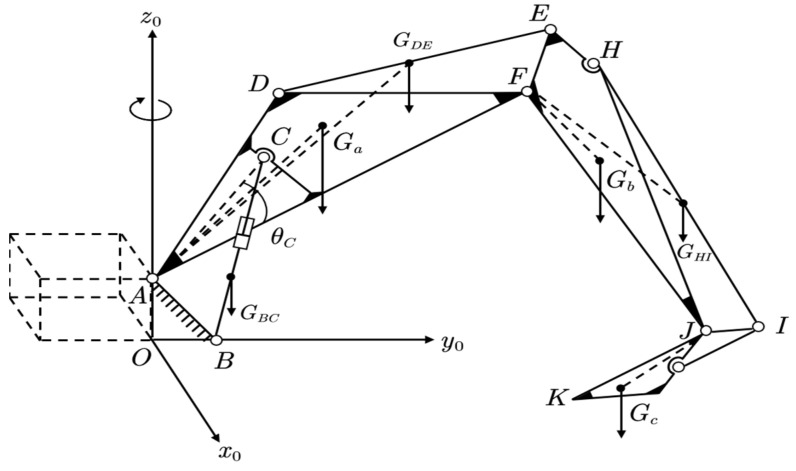
Simplified model of excavator’s upper structure.

For the convenience of subsequent calculations, the excavator’s upper structure was projected onto the symmetrical center plane of the working device to be simplified, as shown in Figure 3.

In the figure above, A~F and H~J are the hinge points between various components of the excavator; K represents the tip of the bucket tooth; $G_{a}$, $G_{b}$, $G_{c}$, $G_{BC}$, $G_{DE}$, and $G_{HI}$ represent the center of gravity positions of the boom, dipper, bucket, and their respective oil cylinders.

**Figure 4:**
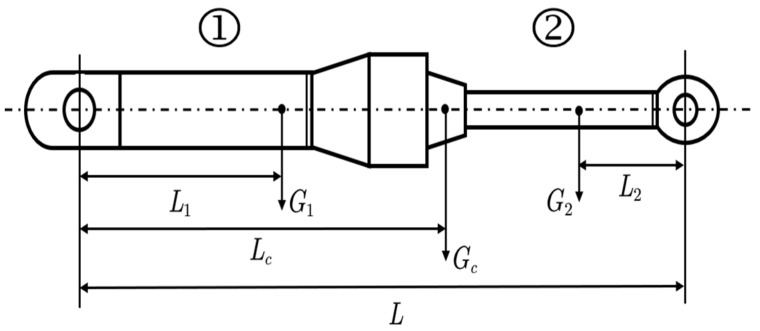
Equivalent model of the composite center of gravity of the working cylinder.

Since the center of gravity position of the cylinder barrel and piston rod of the working cylinder changed with the relative motion of the working device, we needed to simplify its model: the center of gravity position of the cylinder barrel and piston rod was equivalent to a composite center of gravity for calculation. The equivalent model is shown in Figure 4, where ① is the cylinder barrel and ② is the piston rod.

The functional relationship of the composite center of gravity position is as follows:

(1) $$L_{c}=L_{1}+\frac{G_{1}}{G_{2}}\left(L-L_{1}-L_{2}\right)$$

- $G_{1}$ represents the gravity of the cylinder barrel;
- $G_{2}$ represents the gravity of the piston rod;
- $L_{1}$ represents the distance between cylinder and the adjacent hinge point;
- $L_{2}$ represents the distance between piston rod and the adjacent hinge point;
- L represents the distance between two hinge points of the cylinder;
- $L_{c}$ represents the distance between composite center of gravity and hinge point of the cylinder.

The mass parameters of the cylinder barrel and the piston rod of the bucket, dipper, and boom cylinders are obtained by using Solidworks, as shown in Table 2.

The distance from the center of gravity of each working device oil cylinder to its lower connecting hinge point in the initial state is:

$$\begin{array}{ccc} L_{BC}=2629.14 & L_{DE}=3400.55 & L_{HI}=3708.68 \end{array}$$

In which, $L_{BC}$, $L_{DE}$, $L_{HI}$ represent the composite center of gravity of the oil cylinder of the bucket, dipper, and boom, respectively. For each cylinder, the center of gravity coordinates in the initial state can be easily obtained based on their geometric relationship, which is represented as:

(2) $$\left[\begin{array}{c} x_{0}\\ y_{0}\\ z_{0} \end{array}\right]=\frac{L_{c}}{L}\left[\begin{array}{c} x_{BC}\\ y_{BC}\\ z_{BC} \end{array}\right]$$

in which, $x_{BC}$, $y_{BC}$, $z_{BC}$ represent the center of gravity coordinates of the bucket oil cylinders, and the center of gravity coordinates of the dipper oil cylinders and the boom oil cylinders can be calculated similarly. All the initial center-of-gravity coordinates are shown in Table 3.

Ignoring the mass of oil entering and leaving the oil cylinder during the actual operation and only considering the dynamic changes of its center of gravity coordinates, a corresponding D-H transformation coordinate system can be established for calculation.

### 2.2. Calculation of the Center of Gravity Coordinates of Excavator

Excavators’ upper structure can be abstractly viewed as a large engineering manipulator. The most commonly used method for describing their joint motion is the D-H parameter method, which is often used to describe articulated coordinate systems, but for non-joint motion, conventional transformation matrices are necessary. Therefore, the overall transformation matrix for the center of gravity coordinates can be obtained by multiplying the homogeneous transformation matrices determined by the D-H parameter method. In addition, if the excavator itself is not on level ground, the effect of the gravity component on the entire vehicle must be taken into consideration. In this study, we assumed that the initial static state of the excavator was perpendicular to the ground with no obvious tilt or sway [25] and that work scenarios involving uphill or downhill operations are not within the scope of this paper.

#### 2.2.1. Excavator’s Link Coordinate System

**Figure 5:**
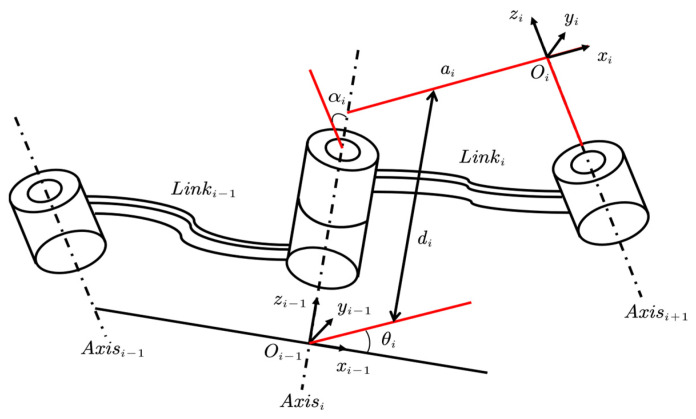
D-H parameters of robotics.

Suppose that two adjacent links in a robot are as shown in Figure 5. We denoted the joint closer to the base as the driving joint and the joint closer to the end effector as the driven joint.

Generally, the D-H coordinate system is established at the driven joint, which is the side closer to the end effector. That is, the coordinate system $\left\{O_{i-1}\right\}$ is fixed to $Link_{i-1}$ and $Axis_{i-1}$ corresponds to the driving axis of the $Link_{i-1}$, $Axis_{i}$ corresponds to the driven axis of the $Link_{i-1}$ and driving axis of $Link_{i}$, $Axis_{i+1}$ corresponds to the driven axis of the $Link_{i}$.

In Figure 5, the two pairs of red lines are parallel to each other:

- $\theta_{i}$—The angle of the x-axis between the coordinate system $\left\{O_{i-1}\right\}$ and the coordinate system $\left\{O_{i}\right\}$, which is also the rotational angle of the $Axis_{i}$;
- $d_{i}$—The offset of the coordinate system $\left\{O_{i}\right\}$ relative to the coordinate system $\left\{O_{i-1}\right\}$ along the $z_{i-1}$-axis, which can be considered as a translation transformation;
- $\alpha_{i}$—The angle between the driving link and the driven link;
- $a_{i}$—The straight-line length between the driving link and the driven link.

**Figure 6:**
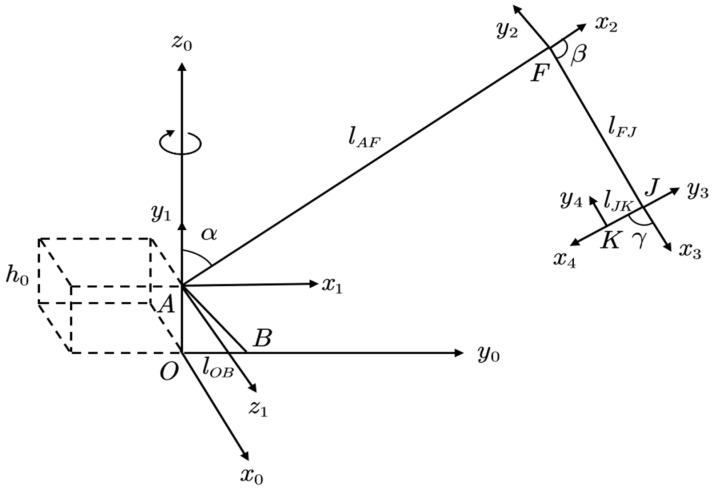
D-H linkage coordinate system of the excavator’s upper structure.

In this context, the rotary coordinate system was fixed to the excavator chassis, and the rotation of the working device relative to their respective pivot points can be considered as the motion of a robot arm with joints. The coordinate system at the boom pivot point A was chosen as the base coordinate system for D-H transformations. Accordingly, the corresponding D-H coordinate systems for the excavator links [26] can be established as shown in Figure 6.

The parameters $l_{AF}$, $l_{FJ}$, and $l_{JK}$ are the distances between the pivot points of the boom, dipper, and bucket, respectively (corresponding to the sizes of the D-H parameters).

#### 2.2.2. The Homogeneous Transformation Matrix of the Excavator

The relevant D-H parameters were determined according to the specific dimensions of the excavator’s upper structure components (links) shown in Figure 6, as shown in Table 4.

According to the parameters in Table 4, the D-H base coordinate system $A-x_{1}y_{1}z_{1}$ can be obtained by rotating and translating the rotary coordinate system $O-x_{0}y_{0}z_{0}$ around the z-axis and x-axis, the corresponding rotation matrix is R10=Rz0(90°)Rx0(90°).

Here, the rotation matrix is determined by Equation (3), and φ is the angle parameter corresponding to this rotation method.

(3) $$R_{z}\left(\varphi \right)=\left[\begin{array}{ccc} \cos\varphi & -\sin\varphi & 0\\ \sin\varphi & \cos\varphi & 0\\ 0 & 0 & 1 \end{array}\right]\;R_{x}\left(\varphi \right)=\left[\begin{array}{ccc} 1 & 0 & 0\\ 0 & \cos\varphi & -\sin\varphi \\ 0 & \sin\varphi & \cos\varphi \end{array}\right]$$

The translation matrix is P10=[00h0]T. Therefore, the homogeneous transformation matrix T10 is:

(4) T10=[R10P10O1]=[00101000010h00001]

Similarly, the reference coordinate system $F-x_{2}y_{2}z_{2}$ can be obtained by rotating and translating the D-H base coordinate system $A-x_{1}y_{1}z_{1}$, with the corresponding rotation matrix as R21 and the translation matrix as P21. The homogeneous transformation matrix T21 is:

(5) T21=[R21P21O1]=[cosα−sinα0lAFsinαsinαcosα0lAFcosα00100001]

By analogy, the transformation matrices between different coordinate systems can be obtained as follows:

(6) T32=[R32P32O1]=[cosβ−sinβ0lFJcosβsinβcosβ0lFJsinβ00100001]

(7) T43=[R43P43O1]=[cosγ−sinγ0lJKcosγsinγcosγ0lJKsinγ00100001]

Continuing from the previous statement, the total homogeneous transformation matrix of the center of gravity of each component relative to the revolving coordinate system can be obtained by multiplying the homogeneous transformation matrices of each coordinate system. Therefore, the total transformation matrices of the joint are:

(8) Point F:T20=T10T21

(9) Point J:T30=T10T21T32

(10) Point K:T40=T10T21T32T43

The 3×1 sub-matrix in the upper-right corner of the total transformation matrix represents the spatial coordinates of the point (${\left[\begin{array}{ccc} x_{i} & y_{i} & z_{i} \end{array}\right]}^{T}$). Combining Figure 3 and Figure 6, it can be seen that the dynamic coordinates of each feature point were difficult to obtain relatively accurately through analytical expressions [27]. Therefore, the dynamic center of gravity of the excavator working device during the rotation process can be obtained by homogeneously transforming its initial center of gravity coordinates.

#### 2.2.3. Calculation of Center of Gravity Coordinates for Each Excavator Module

Let $x_{c}$, $y_{c}$, and $z_{c}$ be the composite center of gravity coordinate of the excavator working device (excluding the corresponding oil cylinder). It was assumed that the mass of each component is uniformly distributed, so the formula for calculating the composite center of gravity is shown as Equation (11):

(11) $$\left[\begin{array}{c} x_{c}\\ y_{c}\\ z_{c} \end{array}\right]=\frac{1}{\sum_{sep=1}^{tol}m_{sep}}\left[\begin{array}{c} \sum_{sep=1}^{tol}m_{sep}x_{s}\\ \sum_{sep=1}^{tol}m_{sep}y_{s}\\ \sum_{sep=1}^{tol}m_{sep}z_{s} \end{array}\right]$$

- $m_{sep}$—The mass of each decomposed module of the excavator (kg);
- tol—Total number of decomposed modules;
- $x_{s},y_{s},z_{s}$—Coordinates of each valid data point.

**Figure 7:**
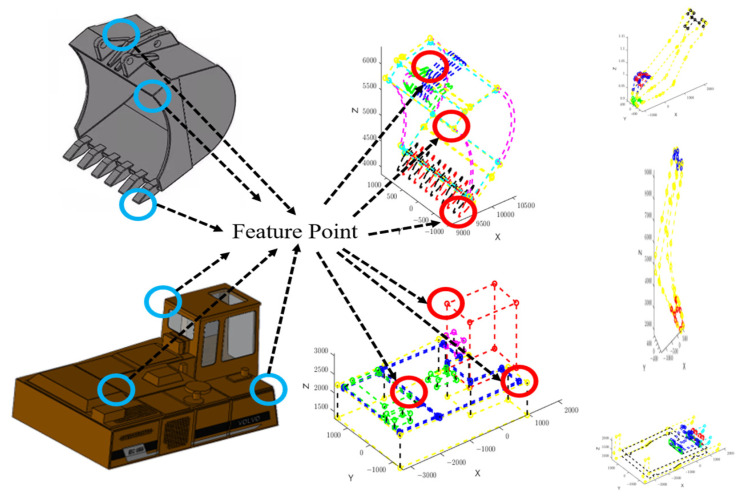
Data restoration of the decomposing module of the excavator.

According to Section 2.1.1, 117 characteristic points were collected for the dipper, 83 characteristic points for the boom, 176 characteristic points for the cockpit, and 168 characteristic points for the base in the initial state. As the bucket is composed of irregular surfaces, the use of linear interpolation would harm the modeling process [28]. Therefore, filling the enclosed area surrounding the bucket surface with additional points can be used as new valid data points [29]. The excavator’s decomposed modules were created by connecting these initial characteristic points, as shown in Figure 7.

**Figure 8:**
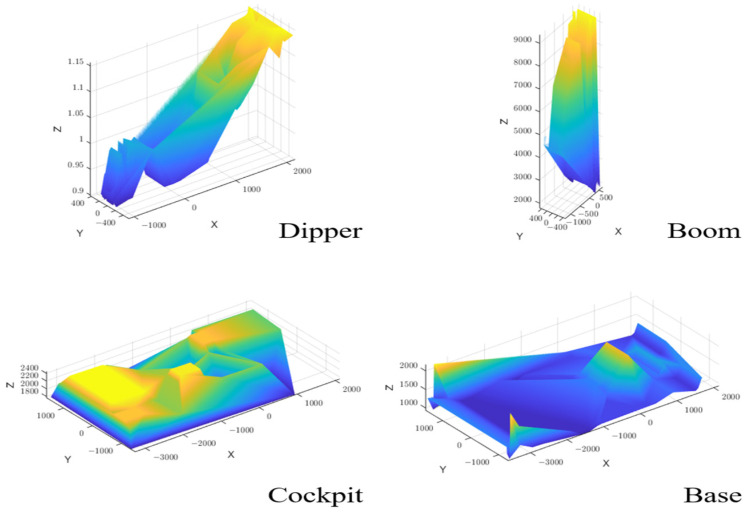
Grid data interpolation fitting results.

Once the initial characteristic points of the 3D model were determined, they were interpolated using the Griddata Method (a method that fits the scattered data points in vector (x,y,z) to a surface v=f(x,y)), and the interpolation results were checked for how well they matched the original model to allow the center of gravity position to be calculated using the expanded valid data points. The interpolation effect is shown in Figure 8, and it is apparent that the expanded valid data points were sufficient to fit the shape of each component.

Combining Equation (11), the center of gravity coordinates of each module of the excavator were calculated based on the set of valid data points and were compared with the measured results from Solidworks software, as shown in Table 5 (where blue represents calculated values, orange represents measured values, yellow represents outliers, and Dv represents the difference).

It can be seen that the calculated values of the center of gravity coordinates of each component of the excavator had some outliers (absolute position error > 30 mm, marked in yellow), which may have been due to the large overall mass of the corresponding component, and the asymmetry in the front, back, left, and right also led to the calculation deviation of the center of gravity coordinates. The algorithm used by Solidworks will make the results more biased toward the symmetrical reference plane of the model, so in actual applications, the characteristics of both methods should be considered to obtain more accurate results. The maximum absolute error between the remaining results and the corresponding software reading values did not exceed 30 mm (excluding the Z-axis, as it is not the sensitive direction of the rotational inertia), which was acceptable relative to the distance of the working device from the vehicle body for several meters. It also had little effect on the final calculation result of the rotational inertia, proving that this method can not only be used to calculate the center of gravity coordinates of engineering vehicles but also overcome various defects in software reading methods and have stronger adaptability.

#### 2.2.4. The Measurement of the Center of Gravity of Excavator’s Load

The maximum bucket capacity of the Volvo EC650 excavator was approximately 3.3 m^3^, and its main targets during excavation operations were soil, sand, and finely crushed rocks. The mixed density of these materials fell within the range of 1.35 to 1.45 g/cm^3^. For the purpose of analysis, we assumed a density of 1.4 g/cm^3^ for the loaded materials and investigated the variation of the excavator’s load center of gravity.

**Figure 9:**
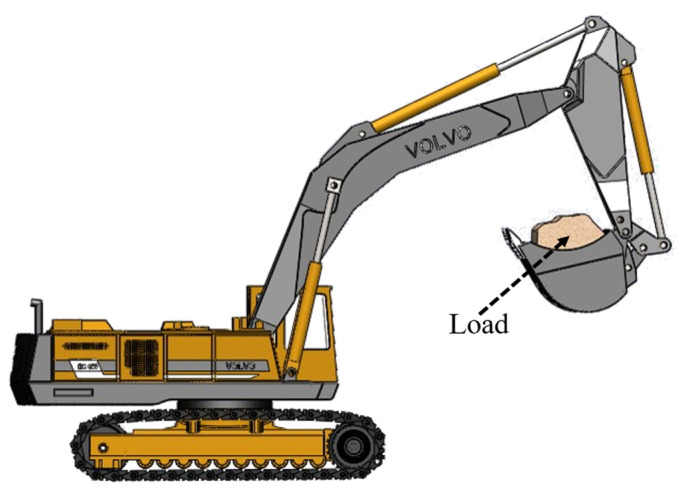
Analysis of excavator load.

In Solidworks, we loaded the excavator bucket with sand and gravel (density set to the aforementioned 1.4 g/cm^3^), and measured the variation of the load center of gravity by increasing and decreasing the volume of sand and gravel (ensuring that the total volume remained within the bucket’s capacity range), as shown in Figure 9.

When the volume of the material reached 3.3 m^3^, the coordinates of the load center of gravity underwent the following changes:

$$X_{Load}=X_{Unload}+1.130\left(\mathrm{mm}\right)\;Y_{Load}=Y_{Unload}-47.387\left(\mathrm{mm}\right)\;Z_{Load}=Z_{Unload}+50.614\left(\mathrm{mm}\right)$$

- $X_{Load}$—The center of gravity of the bucket module when loaded with material;
- $X_{Unload}$—The center of gravity of the bucket module when unloaded.

The representation method for the Y-axis and Z-axis coordinates followed similarly.

**Figure 10:**
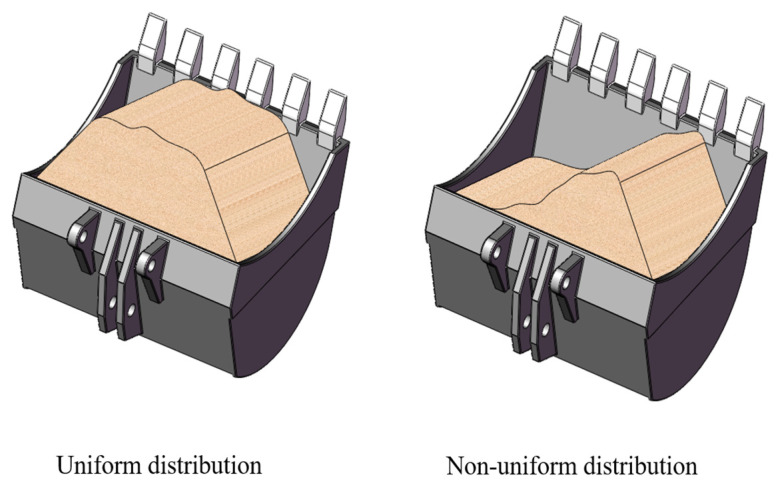
The distribution of material in the bucket.

It is important to note the aforementioned center-of-gravity measurement results assumed that the material was evenly distributed inside the bucket (which is the typical working condition in most cases) and not tilted to one side, as shown in Figure 10.

When the bucket capacity gradually increased, the center-of-gravity variation along the X-axis can be ignored because the even distribution of material did not cause any changes in the center of gravity for a symmetric structure. However, for the variation of the load center of gravity along the Y-axis and Z-axis, it can be calculated using the following formula:

(12) $$\Delta Y=-47.387\cdot \frac{m_{Load}}{4620}=-0.0102m_{Load}\left(\mathrm{mm}\right)\;\Delta Z=50.614\cdot \frac{m_{Load}}{4620}=0.0109m_{Load}\left(\mathrm{mm}\right)$$

- 4620—Total weight at full load.

Indeed, the numerical changes along the Z-axis will not have an impact on the moment of inertia. In the subsequent calculation process, it is sufficient to consider only the variation along the Y-axis.

#### 2.2.5. The Measurement Method of the Center of Gravity of Whole Excavator

Existing excavator’s center-of-gravity measurement techniques mostly focus on measuring the whole vehicle’s center of gravity rather than studying the center of gravity position of individual components separately. This may require data collection during the manufacturing of vehicle components. In the measurement method for the whole vehicle center of gravity, the measurement site is limited by the location of the truck scale, and there are issues such as the excavator having a large tilt angle, which can lead to dangerous situations, significant errors in traditional line-marking methods for center of gravity measurement, and difficulties in obtaining repeatable measurement data. Given the limitations mentioned above, this paper proposed a method for measuring the center of gravity of the entire excavator as follows:

(1) The whole vehicle weight of the excavator M is measured using a truck scale;

(2) The excavator is placed with one side on the knife edge while the other side is suspended with a sling to achieve a horizontal position. A tension detection device is installed on the sling, measure the horizontal distance from the sling to the knife edge, and the value from the tension detection device is recorded. Based on the principle of torque balance, the horizontal distance from the blade edge to the excavator’s center of gravity D is calculated and converted into horizontal coordinates.

(3) When suspending the excavator to an inclined position, measure its tilt angle a and record the value from the tension detection device F. Measure the horizontal distance from the sling to the knife edge d. Then, according to the formula:

(13) Z=[D−(F⋅d)/(M⋅g⋅cos(a))]/tan(a)

Certainly, the vertical distance from the excavator’s center of gravity to the knife edge can be calculated when the excavator is in a horizontal position. This value can then be converted into vertical coordinates.

(4) In step (2), when ‘one side of excavator’ refers to the rear side, the measurement is the horizontal distance $D_{x}$, the tension detection device gives the value $F_{x}$, and the calculated center of gravity coordinates is $X=F_{x}\cdot D_{x}/\left(M\cdot g\right)$; when ‘one side of excavator’ refers to the left side, In the end, corresponding values of $D_{y}$, $F_{y}$, and $Y=F_{y}\cdot D_{y}/\left(M\cdot g\right)$ will be obtained.

**Figure 11:**
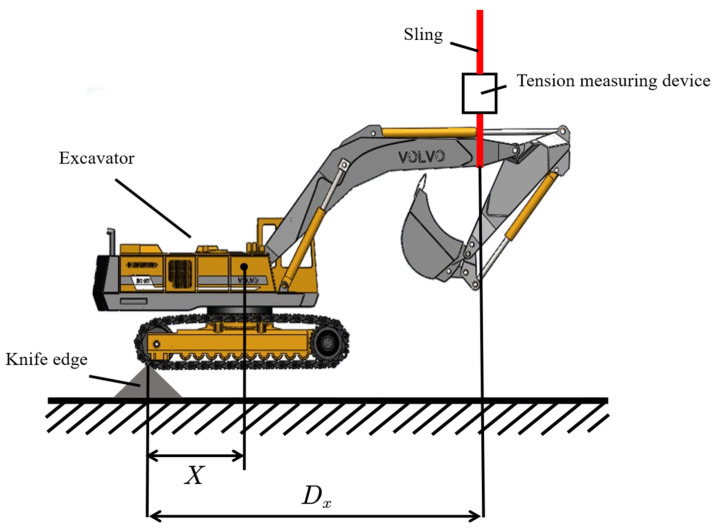
Method of measuring center of gravity of excavator.

A graphical illustration of the above measurement steps is shown in Figure 11.

### 2.3. Calculation of the Rotational Inertia for Excavator

The attitude of the working device of the excavator constantly changed during the actual working process, such as excavation, lifting, rotation, unloading, and other movements or adjustments, which changed the overall vehicle’s rotational inertia [30]. In addition, due to the large distance between the working devices and the rotation center during the working process, coupled with the mass of the rotation platform itself, the excavator as a whole had large rotational inertia. In order to study the influence of the rotational inertia on the braking rotation angle of the excavator, a functional relationship between the rotational inertia relative to the swing axis $O_{Z_{0}}$ of the excavator’s upper structure and the angle of the boom α, the angle of the dipper β, and the angle of the bucket γ was established based on the simplified structure and D-H coordinate system of the upper structure.

(14) $$J_{E}\left(\alpha ,\beta ,\gamma \right)=J_{wk}+J$$

The above equation decomposes the rotational inertia of the entire excavator into two parts: the rotational inertia relative to the axis of each component of the working device, which changes with the position of the center of gravity, and the rotational inertia relative to its axis of the rotation platform during operation, which changes less with rotation.

#### 2.3.1. Calculation of Center-of-Gravity Coordinates for Each Excavator Module

**Figure 12:**
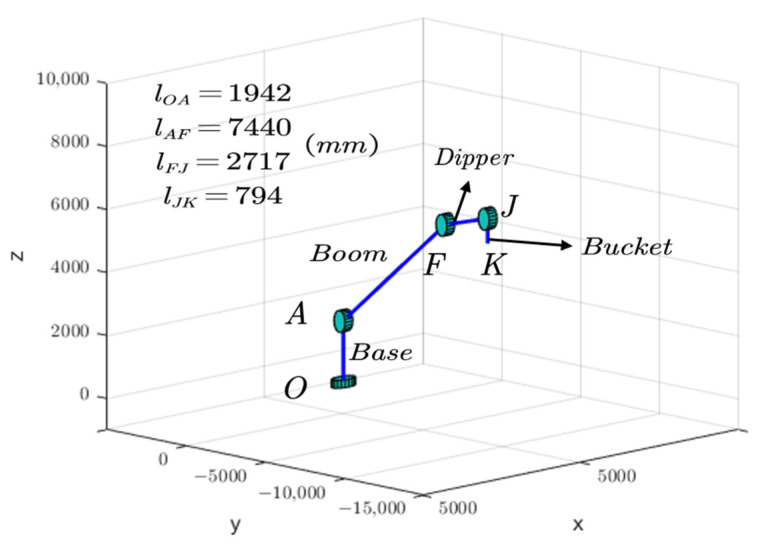
Matlab simulation of the excavator’s upper structure.

Before calculating the rotational inertia of the boom, dipper, and bucket, their working space should be first determined. With the help of Figure 3, a simplified model of the excavator’s upper structure was established using Matlab 2019b software, as shown in Figure 12.

**Figure 13:**
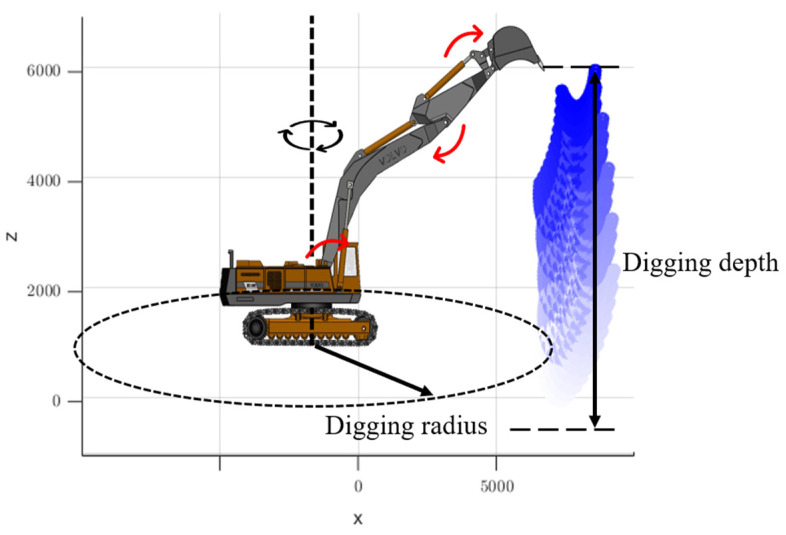
Matlab calculation of the excavator working space.

According to the stroke of each cylinder of the excavator, the range of motion of the boom was determined to be 60~80°, the range of motion of the dipper was 20~75°, and the range of motion of the bucket was 20~150°. The actual rotation range of the entire vehicle was 360°, but the rotational inertia was not affected by the rotation of the vehicle around the slewing axis. To facilitate demonstration, some discrete values were taken as an example. Assuming that the working device of the excavator rotates around the axis 5° each time, we recorded the spatial coordinates of the points that the bucket teeth could reach. There were a total of 270 points, as shown in Figure 13 (The red arrows indicate the direction of rotation of each axis and the black arrow indicates the working radius).

When the excavator performed rotation alone, the distance between each working device and the slewing center remained unchanged, so the rotational inertia of each component did not change. According to the parallel axis theorem for rotational inertia, the rotational inertia of the working device relative to the slewing axis $O_{Z_{0}}$ can be calculated as follows [31]:

(15) $$J_{wk}=\sum_{i=1}^{n}m_{i}d_{i}^{2}+\sum_{i=1}^{n}J_{gi}$$

- $m_{i}$—The mass of each component of the working device (kg);
- tol—The distance from the center of gravity of each component to the slewing axis (mm);
- n—The number of components and hydraulic cylinders in the working device.

The variable $d_{i}$ in Equation (15) can be calculated using the following equation. The calculation method for the related distances in the subsequent equations is the same and will not be repeated here.

(16) di=(Tji(1,4))2+(Tji(2,4))2

Here, Tji(1,4) is the value in the first row and fourth column of the corresponding homogeneous transformation matrix of the component, which represents the value of the center of gravity coordinate x. Similarly, Tji(2,4) represents the value of the center of gravity coordinate y. In addition, $J_{gi}$ is the rotational inertia of each component relative to its center of gravity. Since this part of the inertia does not change much with the working posture of the component, it can be assumed that the rotational inertia changes linearly from the extreme lifting posture to the extreme lowering posture, and a function for $J_{gi}\left(\alpha ,\beta ,\gamma \right)$ can be established as follows:

(17) $$J_{gi}\left(\alpha ,\beta ,\gamma \right)=\Delta J_{i}\times \frac{\varphi^{\prime }}{\varphi }$$

- $\Delta J_{i}$—The total change in rotational inertia of the component from the extreme lifting posture to the extreme lowering posture $\left(\mathrm{kg}\cdot m^{2}\right)$;
- $\varphi^{\prime }$—The rotational angle of each component relative to the hinge point during the working process, where α, β, and γ represent the rotational angles of the boom, dipper, and bucket, respectively (°);
- φ—The range of motion for each component (°).

**Figure 14:**
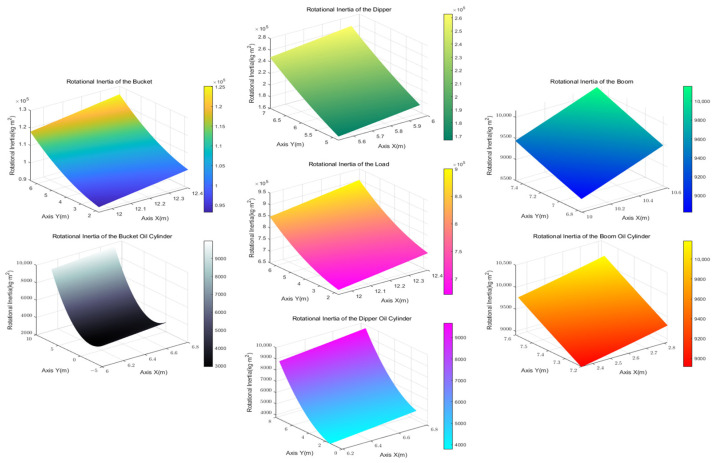
Rotational inertia of excavator working device relative to slewing axis.

It was assumed that the composite center of gravity of each working device cylinder is iteratively obtained from the center-of-gravity coordinates in the initial state. Therefore, the rotational inertia of the bucket, dipper, boom, and their respective cylinders relative to the slewing axis can be calculated according to Table 2 and Table 5, and Equations (15) and (16), as shown in Figure 14.

**Figure 15:**
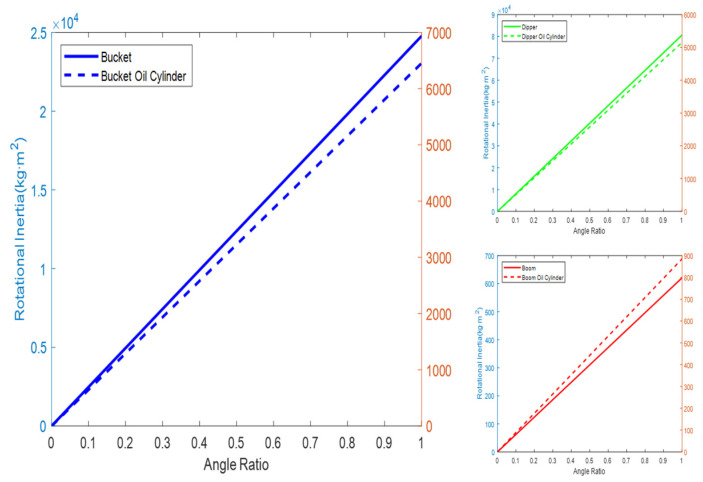
Rotational inertia of the excavator working device relative to its center of gravity.

According to Equation (17), the rotational inertia of the bucket, dipper, boom, and their respective oil cylinder relative to their center of gravity was calculated, as shown in Figure 15.

#### 2.3.2. Calculation of the Rotational Inertia of the Rotation Platform

Since the composite center of gravity of the rotation platform almost does not change in the rotating space, the rotational inertia of the rotation platform is taken as a fixed value:

(18) $$J_{pm}=\sum_{j=1}^{m}m_{j}d_{j}^{2}+\sum_{j=1}^{m}J_{gi}$$

- $m_{j}$—The mass of each component of the rotation platform (kg);
- $d_{j}$—The distance from the center of gravity of each component to the slewing axis (mm);
- m—The number of components in the rotation platform.

The term $J_{gj}$ is similar to $J_{gi}$, which refers to the rotational inertia of each component of the rotation platform relative to its center of gravity (the rotation platform’s center of gravity does not coincide with the slewing axis). This value can be taken as a fixed proportion of the excavator’s overall rotation inertia. Generally, this coefficient is assumed to be $k_{p}=0.1\sim 0.2$.

According to Table 5 and Equation (18), by substituting the mass of the cockpit and the rotation platform as well as their respective distances from the slewing axis, let $k_{p}=0.15$, the rotational inertia of the rotation platform can be calculated $\left(J_{pm}=14239\left(\mathrm{kg}\cdot m^{2}\right)\right)$.

#### 2.3.3. Calculation of the Rotational Inertia of the Excavator

During the actual operation, the mass and mass distribution of each component of the excavator did not change (excluding fuel). The change in the rotational inertia of the whole excavator was caused by changes in the slewing angle of the boom, dipper, and bucket. By combining the previous analyses of the rotational inertia of each component and summing them, the following functional relationship can be obtained:

(19) $$J_{E}=m_{Bucket}\cdot R_{Bucket}+m_{Dipper}\cdot R_{Dipper}+m_{Boom}\cdot R_{Boom}+m_{Load}\cdot R_{Load}+m_{Bucket\;Cylinder}\;\cdot R_{Bucket\;Cylinder}+m_{Dipper\;Cylinder}\cdot R_{Dipper\;Cylinder}+m_{Boom\;Cylinder}\cdot R_{Boom\;Cylinder}+14239$$

In Equation (19):

$$R_{Unload}=7.44s_{\alpha }+2.72\left(c_{\alpha }c_{\beta }-s_{\alpha }s_{\beta }\right)+2.75\left[\begin{array}{c} c_{\gamma }\left(c_{\alpha }c_{\beta }-s_{\alpha }s_{\beta }\right)\\ -s_{\gamma }\left(c_{\alpha }s_{\beta }+c_{\beta }s_{\alpha }\right) \end{array}\right]+0.03\left[\begin{array}{c} c_{\gamma }\left(c_{\alpha }s_{\beta }+c_{\beta }s_{\alpha }\right)\\ +s_{\gamma }\left(c_{\alpha }c_{\beta }-s_{\alpha }s_{\beta }\right) \end{array}\right]\;R_{Bucket}=R_{Unload}^{2}+147.98\;R_{Load}={\left(R_{Unload}-0.047\cdot \frac{m_{Load}}{4620}\right)}^{2}+147.98\;R_{Dipper}=\left\{{\left[7.44s_{\alpha }+2.32\left(c_{\alpha }c_{\beta }-s_{\alpha }s_{\beta }\right)+0.01\left(c_{\alpha }s_{\beta }+c_{\beta }s_{\alpha }\right)\right]}^{2}+32.92\right\}\;R_{Boom}=\left[{\left(0.53c_{\alpha }+7.50s_{\alpha }\right)}^{2}+105.70\right]\;R_{\begin{array}{c} Bucket\\ Cylinder \end{array}}=\left({\left\{\begin{array}{c} -7.44s_{\alpha }+2.72\left(s_{\alpha }s_{\beta }-c_{\alpha }c_{\beta }\right)+7.19\left[\begin{array}{c} s_{r}\left(c_{a}s_{\beta }+c_{\beta }s_{\alpha }\right)\\ -c_{r}\left(c_{\alpha }c_{\beta }-s_{\alpha }s_{\beta }\right) \end{array}\right]\\ +0.05\left[\begin{array}{c} s_{\gamma }\left(c_{\alpha }c_{\beta }-s_{\alpha }s_{\beta }\right)\\ +c_{\gamma }\left(c_{\alpha }s_{\beta }+c_{\beta }s_{\alpha }\right) \end{array}\right] \end{array}\right\}}^{2}+40.92\right)\;R_{\begin{array}{c} Dipper\\ Cylinder \end{array}}=\left\{{\left[-7.44s_{\alpha }+0.03\left(c_{\alpha }s_{\beta }+c_{\beta }s_{\alpha }\right)+6.51\left(s_{\alpha }s_{\beta }-c_{\alpha }c_{\beta }\right)\right]}^{2}+42.24\right\}\;R_{\begin{array}{c} Boom\\ Cylinder \end{array}\cdot }=\left[{\left(1.53c_{\alpha }+7.43s_{\alpha }\right)}^{2}+6.64\right]$$

**Figure 16:**
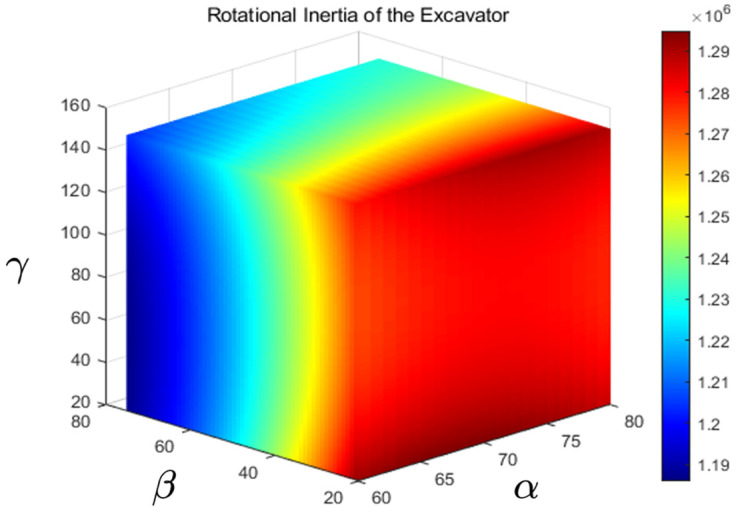
The rotational inertia of the excavator’s upper structure.

$m_{Bucket}$, $m_{Dipper}$, $m_{Boom}$, $m_{Load}$, $m_{Bucket\;Cylinder}$, $m_{Dipper\;Cylinder}$, and $m_{Boom\;Cylinder}$ represent the mass of the bucket, dipper, boom, load, bucket cylinder, dipper cylinder, and boom cylinder, respectively; $s_{\alpha }$, $c_{\alpha }$, $s_{\beta }$, $c_{\beta }$, $s_{\gamma }$, and $c_{\gamma }$ represent the sinα, cosα, sinβ, cosβ, sinγ, and cosγ respectively. According to Equation (19), the relationship between the rotational inertia of excavator $J_{E}$ and the variations in α, β, and γ can be graphically represented as shown in Figure 16.

### 2.4. Calculation of the Braking Rotation Angle

The braking rotation angle of an excavator refers to the angle when the vehicle runs at the maximum rotation speed and starts braking by cutting off the motor oil supply until the speed reaches zero. Currently, there is no effective method for accurately calculating the exact value of this angle, because it is difficult to accurately measure the excavator’s rotational inertia and output torque [32].

#### 2.4.1. Analysis of the Excavator Rotation Motion

**Figure 17:**
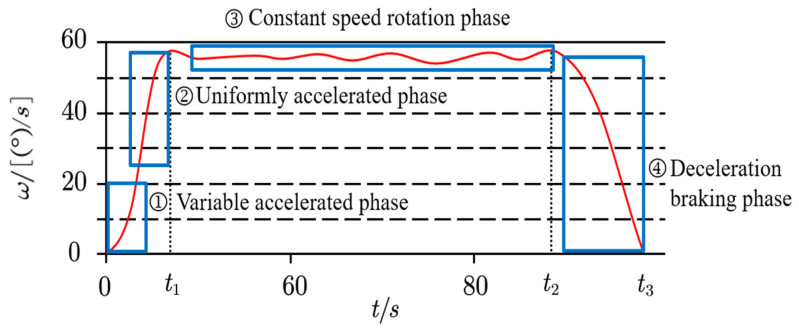
The rotary characteristic curve of the excavator.

As mentioned earlier, the initial braking position of the excavator is at the horizontal plane, and its rotation system is a large inertial system, so its rotary motion characteristics determine the braking angle of the entire vehicle [33]. When the rotation motor and the working oil cylinder perform a combined action, the rotation process can be divided into three stages: the starting acceleration stage, the constant speed slewing stage, and the deceleration braking stage. Among them, the acceleration and deceleration stages have a greater impact on the braking angle. Therefore, considering the torque output of the hydraulic system during these two stages can more accurately predict the excavator’s braking rotation angle. The rotary motion characteristic curve of the Volvo EC650 excavator was plotted by collecting angular velocity data [34], as shown in Figure 17.

From Figure 17, it can be seen that the $0\sim t_{1}$ segment is the start-up acceleration phase of the excavator’s rotary platform, which can be further divided into a variable acceleration phase and a uniform acceleration phase, and they can be simplified as a uniform acceleration process for simplicity. $t_{1}\sim t_{2}$ segment is the uniform rotation phase, during which the excavator enters this phase when the speed of the rotation platform reaches its maximum, and its speed–time curve approximates a trapezoid. $t_{2}\sim t_{3}$ segment is the deceleration braking phase, during which the speed of the excavator gradually decreases to zero and generates a braking rotation angle. Typically, the state in which the excavator begins to brake is referred to as the “stalled state”, meaning that the excavator’s kinetic energy is gradually consumed until it comes to a complete stop, which occurs when the brake pedal is pressed or the throttle pedal is released.

#### 2.4.2. Typical Characteristics Analysis of Excavator Hydraulic System

**Figure 18:**
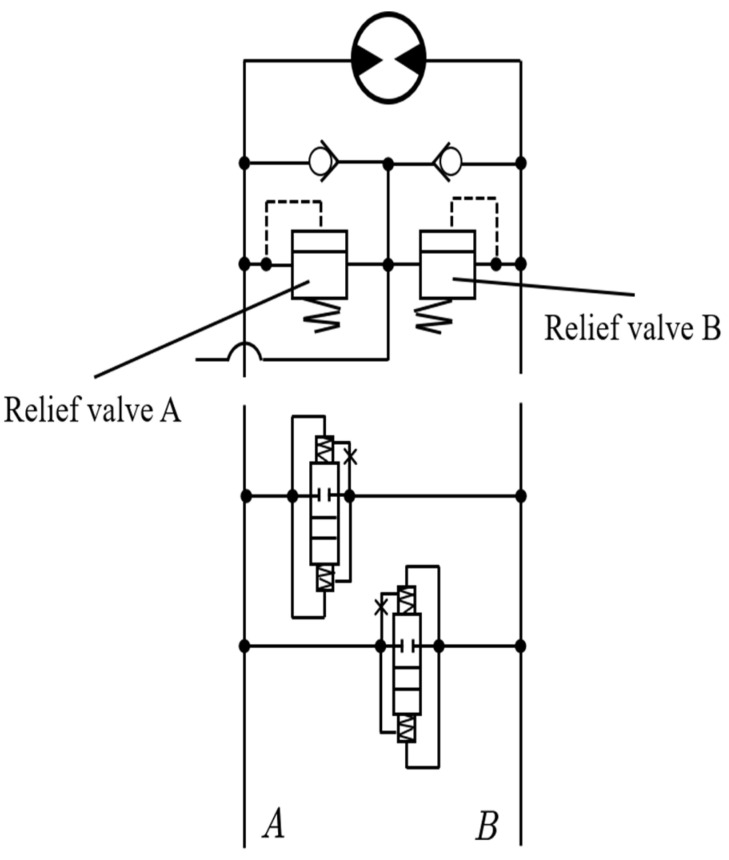
Rotary motor hydraulic schematic.

The hydraulic excavator performs hydraulic braking through the motor when braking rotation. The hydraulic principle of the rotary motor is illustrated in Figure 18. Assuming the reducer rotates in a certain direction, high-pressure oil is supplied to port A while low-pressure oil returns to port B. When braking, the hydraulic circuit from the hydraulic pump is cut off, i.e., ports A and B are blocked. As a result, the rotary motor operates in a pump mode under the influence of the inertia torque of the turntable. The oil pressure at port A decreases while the pressure at port B increases until it reaches the set pressure of the relief valve B. Consequently, the relief valve B opens, allowing the overflow to flow back to port A.

Calibrating an accurate model for hydraulic braking equipment involves gathering a substantial amount of data, which can be expensive and time-consuming. Additionally, it is challenging to obtain a pure physical model that suits different behaviors of various braking equipment by precisely modeling each individual component. This is primarily due to the following issues [35]:

(1) For fluid systems, hydraulic equipment consists of components that exhibit highly nonlinear behavior, and these components often generate strong discontinuities in the solutions. Moreover, the nonlinear behavior is not only influenced by the values of the considered system states (such as pressure and flow rate) but also by their derivatives. This type of behavior is particularly common in electromechanical and electro-hydraulic actuators [35].

(2) Internal friction affects the response of the brake in terms of applied torque/force. In practical operation, both friction and more general hysteresis effects are strongly influenced by the travel speed [36].

To reduce computational load, certain parameters involved in the calculations can be simplified. During the braking process, the pressure at port B remains constant at the set pressure of the relief valve B (also known as constant-pressure relief). Referring to Figure 17, during the acceleration phase $0\sim t_{1}$, the pressure at port A rapidly increased and then remained almost constant at high pressure (i.e., constant-pressure relief), while the pressure at port B almost reached zero. When the speed is constant in a certain time period $t_{1}\sim t_{2}$, the pressures at ports A and B are nearly zero. In the braking phase of the time period $t_{2}\sim t_{3}$, the pressure at port B rapidly rises and then remains at constant high pressure, while the pressure at port A remains at low pressure. By considering the acceleration and deceleration phases $0\sim t_{1}$ and $t_{2}\sim t_{3}$ as uniform acceleration and uniform deceleration motion, with equal magnitude and opposite direction of acceleration, according to the torque formula M=J×ω (where M is the torque (N⋅m), J is the rotational inertia $\left(\mathrm{kg}\cdot m^{2}\right)$, and ω is the angular acceleration $\left(\mathrm{rad}/s^{2}\right)$), the braking torque is equal in magnitude to the rotational torque, enabling further calculations.

#### 2.4.3. Prediction of the Braking Rotation Angle

When the excavator needs to change its posture during the rotation process, the rotational inertia of the whole vehicle is not fixed. Therefore, the continuous function for calculating the braking angle needs to be discretized, and the time axis is divided into finer intervals for integration [34]. When the excavator does not have any additional actions during the rotation process, the rotational inertia of the whole vehicle is constant. It was previously known that the rotational torque and the braking torque are the same magnitude, then:

(20) $$M_{B}=M_{W}=M_{R}\times \rho_{s}\times i_{s}$$

- $M_{B}$—The braking torque of excavator (N⋅m);
- $M_{W}$—The rotating torque of excavator (N⋅m);
- $M_{R}$—The output torque of reducer (N⋅m);
- $\rho_{s}$—The transmission efficiency of swing bearing;
- $i_{s}$—The transmission ratio from the output gear of the reducer to the swing bearing shaft.

Predicting the braking rotation angle by combining Equation (20) and acceleration formula:

(21) $$\theta =\frac{180}{\pi }\times \frac{1}{2}\times \omega_{B}\times {\left(\frac{\omega_{\max}}{\omega_{B}}\right)}^{2}=\frac{90}{\pi }\times \frac{\omega_{\max^{2}}J_{E}}{M_{B}}$$

- θ—The braking rotation angle (°);
- $\omega_{B}$—The angular acceleration during the braking process $\left(\mathrm{rad}/s^{2}\right)$;
- $\omega_{\max}$—The maximum rotation speed of excavator (rad/s).

In Equation (21), $\omega_{\max}=\frac{\omega_{R}}{i_{s}}$, $\omega_{R}$ is the operating rotation speed of the reducer (rad/s). By combining Equations (20) and (21), we can obtain:

(22) $$\theta =\frac{90}{\pi i_{s}^{3}\rho_{s}M_{R}}\times J_{E}\omega_{R}^{2}=\lambda \times \frac{J_{E}\omega_{R}^{2}}{M_{R}}\left(\lambda =\frac{90}{\pi i_{s}^{3}\rho_{s}}\right)$$

In general, the transmission efficiency of excavator slewing support $\rho_{s}$ is above 90%, while the transmission ratio from the output gear of the reducer to the slewing support shaft $i_{s}$ may vary depending on the model and manufacturer. Usually, this ratio is 3~6. Therefore, in a specific excavator, the selection of the transmission ratio needs to be determined based on its working load, speed, torque, and other parameters. For the same model (EC650) of the excavator, if $\rho_{s}=90\%$ and $i_{s}=4.5$ are given, then λ can be considered a constant. The predicted braking rotation angle θ is positively correlated with the excavator’s rotational inertia $J_{E}$ and the square of the working speed of the reducer $\omega_{R}$, and negatively correlated with the working output torque of the reducer.

The output torque of the reducer $M_{R}$ is generally determined according to Equation (23).

(23) $$M_{R}=9550\times \frac{P_{M}}{\omega_{M}}\times i_{R}\times \xi$$

- 9550—The torque calculation formula coefficient;
- $P_{M}$—The real-time engine power (kw);
- $\omega_{M}$—The engine output speed (r/min);
- $i_{R}$—Gear ratio, which is the ratio of engine output speed to the output speed of the gearbox;
- ξ—The gearbox mechanical efficiency.

Substituting Equation (23) into Equation (22), we obtain:

(24) $$\theta =\frac{\lambda \cdot \omega_{M}\cdot \omega_{R}^{2}\cdot J_{E}}{9550\cdot i_{R}\cdot \xi \cdot P_{M}}$$

**Figure 19:**
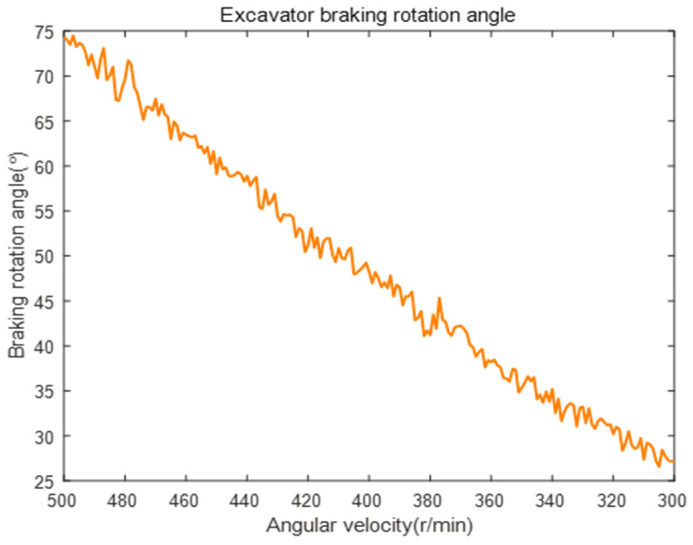
The braking rotation angle of the excavator in a stagnation state.

Before entering the deceleration and braking process, the excavator’s engine and gearbox operate stably, allowing the entire vehicle to enter the “idle rotation” state, during which the speed of the gearbox gradually decreases from its initial value to its minimum value. Consult the relevant manual and select the parameters corresponding to the excavator under full load as follows: $P_{M}=393$, $\omega_{M}=1800$, $i_{R}=9600$, ξ=0.83, and $\omega_{M}=600\sim 800$. However, these parameters will not remain fixed during the rotation process but will fluctuate within a certain range, which will result in a disturbance factor Δ in Equation (24). The mean value of the $\omega_{R}$ was taken as 700 and Δ conformed to the normal distribution. The excavator’s braking rotation angle change relationship under the stationary state was calculated by substituting random numbers, as shown in Figure 19.

From Figure 19, it can be seen that although the braking rotation angle fluctuated continuously as the reducer speed decreased, it will gradually decrease to its minimum value. To more scientifically evaluate the braking performance of the excavator, further consideration was given to how to offset most of the effects caused by the difference in rotation speed and torque. According to Equation (25), the theoretical values of the excavator’s rotation speed $\omega_{E}$ and rotation torque $M_{E}$ are represented by $\omega_{Et}$ and $M_{Et}$, respectively, which are calculated as:

(25) $$\omega_{Et}=\frac{f_{\max}\rho_{v}}{Vi_{s}i_{R}}\times 10^{3}\;M_{Et}=\frac{PVi_{s}i_{R}}{2\pi }\rho_{M}\rho_{s}\xi$$

- $f_{\max}$—The maximum flow rate of the swing motor (L/min);
- $\rho_{v}$—The volumetric efficiency of the swing motor (mL/r);
- $P_{M}$—The mechanical efficiency of the swing motor;
- V—The displacement of the excavator (mL/r);
- P—The working pressure of the swing motor (MPa).

Generally speaking, the maximum flow rate of the swing motor for medium-sized excavators is around 30~60(L/min), while for large excavators, the maximum flow rate may reach 100(L/min) or even higher. In addition to the maximum flow rate, the volumetric efficiency of the swing motor is also a very important indicator, which refers to the ratio of the flow rate to the speed of the swing motor during operation. It affects the rotation speed and output power of the excavator and usually ranges from 0.8~2.0(mL/r). Furthermore, the mechanical efficiency of the swing motor refers to the ratio of the mechanical power output to the hydraulic power input, which is directly related to the energy consumption of the excavator, and is generally around 80~90%. The excavator’s displacement refers to the amount of oil output by the excavator per unit of time, usually expressed by (mL/r), and the size of the displacement determines the torque of the entire vehicle, generally ranging from 10 to 100 (mL/r). Finally, the working pressure of the swing motor refers to the pressure of the hydraulic oil supplied by the hydraulic system to the swing motor, which is measured in MPa, and an appropriate value is around 10~35(MPa).

## 3. Results

Therefore, selecting $f_{\max}=45\left(L/\min\right)$, $\rho_{v}=1.4$, $\rho_{M}=85\%$, V=90(mL/r), and P=30(MPa), the predicted braking rotation angle was corrected according to Equation (26):

(26) $$\theta^{\prime }=\theta \times \frac{{\left(\omega_{Et}/\omega_{E}\right)}^{2}}{M_{Et}/M_{E}}$$

- $\omega_{E}$—Actual rotation speed of excavator (r/min);
- $M_{E}$—Actual rotation torque of excavator (N⋅m).

**Figure 20:**
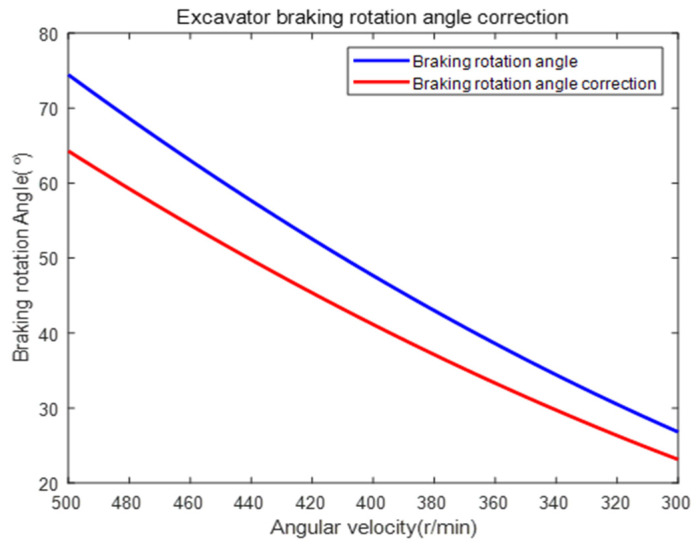
Correction of the excavator braking rotation angle.

Substituting $\omega_{E}=\frac{\omega_{R}}{i_{R}}$ and $M_{E}=M_{W}$ into Equation (26), compare θ and $\theta^{\prime }$ before and after correction, as shown in Figure 20.

Based on Figure 20, the comparison between the braking rotary angles before and after the correction showed a numerical difference of 13.67% (i.e., the corrected angle was 13.67% different from the angle before correction). However, there is currently no clear reference range for the braking rotation angle of excavators, and the precise values of the vehicle’s rotational inertia and output torque cannot be obtained [30]. Therefore, it is necessary to complete the vehicle coordination in actual application scenarios, collect key parameters that reflect its ability status, and use reinforcement learning to make the predicted braking angle more reasonable.

**Figure 21:**
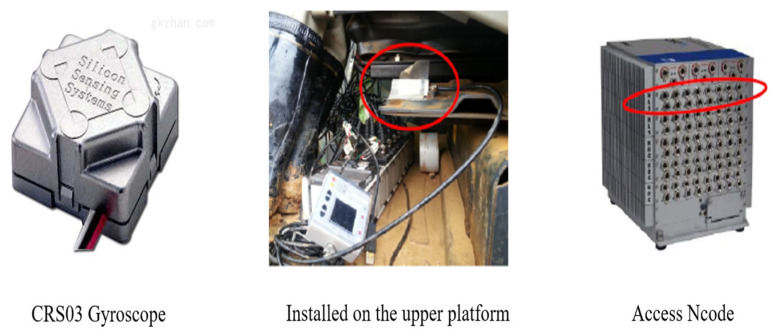
Experimental instrument.

The instrument used to measure the braking rotation angle is shown in Figure 21.

The angular velocity sensor (CRS03 gyroscope) was fixed at a certain position on the upper platform of the excavator, and its output signal was connected to the Ncode high-low level layer. The measurement range was ±100(°)/s (turning right means positive, and turning left means negative), and resolution was 0.05(°)/s.

When conducting the test for the braking rotary angle, it was necessary to quickly operate the pilot control lever to its maximum stroke, causing the excavator to rotate at its highest speed. When the excavator completed one full rotation and returned to the starting point (this process requires assistance from personnel observing and signaling to the operator), the pilot control lever was swiftly released to its neutral position, allowing the excavator to naturally brake and come to a stop. Then, the data collected by Ncode can be processed through integration to obtain the braking rotary angle.

Table 6 shows the data obtained from measuring the braking rotary angle of a batch of the same model (50-ton class hydraulic excavator) from a certain company.

In theory, the braking rotary angle for the same model should be the same. However, the measured angle difference rate was as high as 19%. After analysis, the potential reasons for this discrepancy may include variations in the rotary speed, differences in rotary torque, the positioning of the excavator during the experiment, operator’s actions, mechanical efficiency, and reading accuracy, all of which can influence the predicted results [37].

It was obvious that the predicted whole vehicle braking rotary angle after correction was closer to the measured value (the average corrected braking rotary angle for 8 excavators in the experiment was 61.401(°), while the predicted braking rotary angle when the excavator starts braking and the reducer reached maximum speed was 64.288(°)). This demonstrates that the correction method can compensate for some of the differences in rotary speed and torque, validating the scientific and effective nature of the method proposed in this paper. The method is also applicable for comparing prototypes with benchmarks. When manufacturing companies upgrade their products based on market demands, the excavator’s rotary speed and torque may undergo changes. In such cases, this method can be utilized to predict the whole vehicle braking rotary angle after the upgrade to evaluate its braking performance. For instance, if it is determined that the braking is too fast, adjustments can be made by increasing the maximum rotary speed or reducing the whole vehicle’s rotary torque.

**Figure 22:**
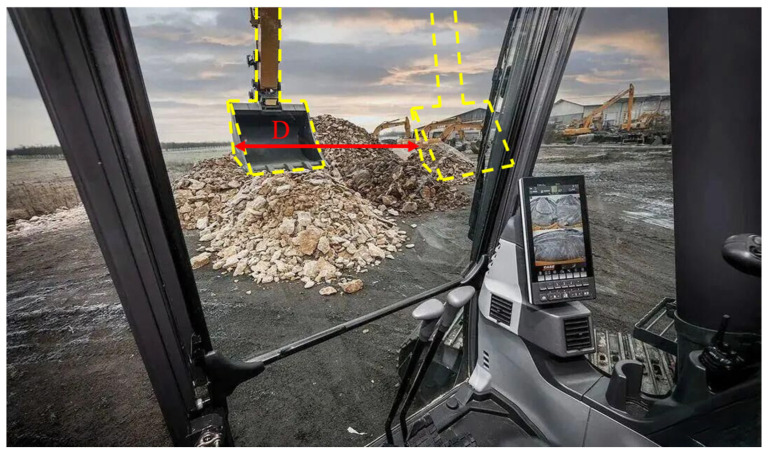
Application scenario of the excavator braking rotation angle.

Figure 22 shows an another application scenario of the excavator braking rotation angle, where people can determine the distance D between the final position of the bucket and the current position based on the calculated value and the distance between the bucket and the cockpit, providing visual assistance for the remote-controlled operation of construction machinery.

## 4. Discussion

The simulation result showed that the excavator had a simplified and clear structure, and the calculation model of the center of gravity and rotational inertia was accurate and reliable. The predicted value of braking rotation angle was in good agreement with the experimental value. In actual application, the prediction frequency can be determined by the gradually reducing speed of the gearbox during the rotation braking process, and the use of multiple dynamic corrections can ensure that the predicted position is consistent with the final stopping position. This result also proved that the prediction of the excavator’s braking rotation angle based on the dynamic change of the rotational inertia is feasible. In engineering applications, calibration or learning modes can be introduced to optimize certain parameters selected in the entire method and improve the final prediction accuracy.

The methods and problem-solving strategies employed in this study can also be applied to the prediction of other actions of remote-controlled excavators and related research on the structures of similar large-scale construction machinery. With the acquisition of relevant parameters α, β, and γ, the dynamic changes in the rotational inertia during the rotation process of the vehicle at any posture can be analyzed. Combining this with the performance parameters of the gearbox, the final braking rotation angle after the rotation process can be predicted, providing a reliable solution for remote-controlled operations of construction machinery.

We hope to conduct the following experiments or solve the following problems in the future:

(1) In subsequent research, when conditions permit, it would be advantageous to choose specific components of the excavator and measure their actual center of gravity positions. These measured data can then be compared with the results obtained from the simulation method proposed in this paper. It is important to note that the center-of-gravity position only needs to be measured once, and in subsequent calculations, it can be compared with the values obtained from specific software reading values, allowing for the selection of data that best suits the research needs. Once we obtain the relative position of the center of gravity with respect to the corresponding coordinate system for a particular component, we can conduct homogeneous transformation studies to investigate the dynamic changes in the center of gravity.

(2) The excavator’s load varies with changes in the bucket capacity, and the increase or decrease in oil in each working device’s cylinder can also be considered as part of the overall load of the vehicle. The latter was neglected in this paper. While the former was analyzed to some extent, the calculations were based on full load conditions. In practical applications, it is recommended to employ visual methods to reconstruct a 3D model of the material pile and estimate its volume and mass to improve the accuracy of predictions.

(3) For the braking rotation angle, its influencing factors are concentrated in various stages of the braking rotation process. This involves complex velocity and energy transformations, making accurate modeling and utilization challenging. Combining the model with the hydraulic system could potentially enhance the prediction accuracy and applicability. Many assumptions made in this paper were based on ideal and simplified conditions, and the selection of data requires further discussion and limitations. Once precise control of the vehicle’s rotation speed during the rotation process is achieved, all parameters related to speed can be separated from Equation (24). The entire prediction model can then be divided into constant (determined by the mechanical characteristics of the excavator) and variable (rotational inertia, hydraulic system, rotation speed, etc.) parts for separate calculations.

(4) Currently, there are very limited experimental conditions for testing the braking rotation angle of engineering vehicles. The experimental method presented in this paper effectively approximated the theoretical requirements, and the obtained prediction results were also satisfactory. Subsequent research should focus on certain factors such as errors due to human factors, the applicability of this method to different vehicles, and improvements to the correction formula. It should also promote the construction, maintenance, and development of experimental equipment and facilities.

## Figures and Tables

**Table 1:** Part of the excavator’s upper structure decomposition module initial feature points.

	X(mm)	Y(mm)	Z(mm)
Bucket	9044.76 9176.78	1163.13 −211.28	5707.14 6064.93
	9407.62	1058.87	4086.27
	…	…	…
Dipper	74.74	362.83	8975.42
	356.85	469.49	1799.85
	210.59	−271.24	9210.51
	…	…	…
Boom	1677.87	432.11	11,356.96
	1863.26	−543.72	11,046.83
	−790.45	102.45	9047.63
	…	…	…
Cockpit	937.94	−1466.71	1318.00
	−1941.74	−516.58	2246.00
	2128.74	1524.44	1318.00
	…	…	…
Base	−3455.88	1526.13	928.00
	1691.86	−610.60	1298.00
	909.9	651.99	1064.50
	…	…	…
Bucket Cylinder	6650.60	127.57	7601.44
	9201.53	136.67	6832.21
	6438.83	−16.73	7656.54
	…	…	…
Dipper Cylinder	5392.85	104.42	7365.69
	2775.00	−47.76	5945.77
	2570.43	−49.49	5828.12
	…	…	…
Boom Cylinder	2203.96	455.68	4921.01
	2503.70	436.10	4688.81
	1298.90	379.26	1456.04
	…	…	….

**Table 2:** Quality parameters of the cylinder in each working device of the excavator.

	Bucket Cylinder	Dipper Cylinder	Boom Cylinder
$m_{1}\left(\mathrm{kg}\right)$	47.52	62.27	94.64
$L_{1}\left(\mathrm{mm}\right)$	1093.82	1463.86	1095.12
$m_{2}\left(\mathrm{kg}\right)$	31.52	31.52	61.01
$L_{2}\left(\mathrm{mm}\right)$	901.16	901.16	680.16
L(mm)	3013.36	3345.34	3460.12

**Table 3:** Initial center-of-gravity coordinates of each cylinder.

	$x_{0}$	$y_{0}$	$z_{0}$
Bucket Cylinder	6399.00	54.18	6396.68
Dipper Cylinder	3795.65	32.14	6499.07
Boom Cylinder	1533.37	12.98	2576.92

**Table 4:** Parameters of the linkage of the excavator’s upper structure.

	θ(°)	d(mm)	α(°)	a(mm)
$\left\{O_{0}\right\}\to \left\{A\right\}$	90	$h_{0}=1942$	90	0
{A}→{F}	α	0	0	$l_{AF}=7440$
{F}→{J}	β	0	0	$l_{FJ}=2717$
{J}→{K}	γ	0	0	$l_{JK}=794$

**Table 5:** Comparison of the center-of-gravity coordinates of each module.

	X(mm)	Y(mm)	Z(mm)
Bucket	1955.37	−30.30	12,164.83
640.23(kg)	2049.41	−58.70	12,453.02
DV	94.04	28.40	288.19
Dipper	525.00	−60.00	10,281.00
3110.89(kg)	575.25	−56.93	10,182.78
DV	50.25	3.07	98.22
Boom	−400.00	−1.33	5737.40
60.62(kg)	−412.42	−2.50	5461.14
DV	12.42	1.17	276.26
Cockpit	−570.00	363.60	2187.10
350.48(kg)	−525.21	351.36	2308.20
DV	44.79	12.24	121.10
Base	−1747.30	144.00	1150.50
4104.14(kg)	−1718.95	162.51	1191.53
DV	29.65	18.51	41.03
Σ	−605.40	46.29	2604.01
12,409.66(kg)	−601.69	59.74	2629.02

**Table 6:** Test data for braking rotary angle of 50-ton class excavator.

Serial Number	Braking Rotation Angle/(°)
1	60.05
2	58.12
3	61.39
4	59.53
5	63.80
6	60.13
7	69.25
8	58.94
Maximum value	69.25
Minimum value	58.12
Difference rate	19%
